# Refractory Behçet’s disease treated with low-dose interleukin-2: A case report

**DOI:** 10.1097/MD.0000000000031173

**Published:** 2022-10-21

**Authors:** Wenyan Zhou, Tian Liu, Xian Xiao, Jing He

**Affiliations:** a Department of Rheumatology & Immunology, Peking University People’s Hospital, Beijing, China; b Beijing Key Laboratory for Rheumatism Mechanism and Immune Diagnosis (BZ0135), Beijing, China.

**Keywords:** Behcet’s disease, IL-2, refractory, treatment

## Abstract

Behçet’s disease (BD) is an autoimmune disease presented with recurrent oral aphthous ulcers, genital ulcers, uveitis and skin lesions. The cutaneous manifestations are often refractory and more resistant to conventional therapy. This report provides a new treatment of low-dose interleukin-2 (IL-2) with refractory BD patient.

**Patient concerns:**

A 37-year-old female patient experienced oral ulcer and erythema nodosum on the right leg for over 12 months and resisted to Methylprednisolone and Thalidomide.

**Diagnoses:**

The patient suffered from recurrent painful oral ulceration and an erythema nodosum. Pathergy test is also positive. Thus, we diagnosed her as BD according to the International Criteria for Behçet’s Disease (ICBD).

**Interventions:**

The patient took Methylprednisolone 8 mg qd, Thalidomide 50 mg qn and Hydroxychloroquine 200 mg bid successively as treatment. However, the medicine didn’t take effect. Finally, this patient was given low-dose IL-2 intramuscular injection qod for 3 months.

**Outcomes:**

Oral ulcers and the erythema disappeared and the patient has been symptom-free for 6 months.

**Lessons:**

low-dose IL-2 is a safe and effective treatment for refractory BD.

## 1. Introduction

Behçet’s disease (BD) is a chronic, multisystemic, and recurrent inflammatory disorder of unknown etiology, characterized by recurrent oral aphthous ulcers, genital ulcers, uveitis, and skin lesions, prevalent in the third and fourth decade of life of people of the eastern Mediterranean and eastern Asian.^[1,2]^

An intense neutrophil infiltration in organs has been reported in the early stage of inflammation in BD.^[3]^ Hyperactivation of neutrophil caused macrophage activation, imbalanced Th1 cells and increased frequencies of Th17 cells, giving rise to cutaneous lesions and thrombosis.^[4]^ Innate lymphoid cells (ILCs) are lymphoid cells that have important effector and regulatory functions in innate immunity and tissue remodeling. Gunnur Deniz et al (2021) reported increased total ILC and ILC3 cells in active BD patients, which shed light on the inflammatory microenvironment in BD patients.^[5]^ However, the role of ILC2 cells involved in the development of BD cells hasn’t been reported till now.

Thalidomide and topical corticosteroids have been routine medications for patients of Behçet’s disease, especially for recurrent skin lesions. Some obtain remission or achieve sustained control for oral and genital ulcers.^[6]^ However, for those who fail to respond to this therapy, relapsing because of its severe side effect or refractory disease, with other manifestations on the skin such as erythema nodosum, we should chase for a new treatment. Low-dose interleukin-2 (IL-2) has been successfully used in a variety of autoimmune diseases. In addition, IL-2 has been reported to promote the amplification of Tregs and mitigates clinical manifestation in patients with refractory Behçet’s disease.^[7,8]^ However, to date, there has been no report of the effect of low-dose IL-2 treatment on Behçet’s disease.

Here, we report a patient with refractory Bechet disease who was unable to tolerate Thalidomide. Thus, we treated her with low-dose IL-2 for 3 months and got an unexpected outcome.

## 2. Case presentation

A 37-year-old woman, with no history of infectious disease and chronic medical illness, suffered from recurrent painful oral ulceration for over one year. She didn’t go to the doctor and take any treatment. Recently she discovered that an erythema nodosum about 3 cm in diameter occurred on the front of her right lower extremity with tenderness. The erythema is dark red without a limited boundary, though the epidermis is complete. The patient denies any history of lower limb trauma, allergy or previous leg surgeries.

Laboratory tests showed no abnormal results of C reactive protein, erythrocyte sedimentation rate, and the liver and kidney function is also in the normal range. HLA-B51 and other antinuclear antibodies are negative, except anti-endothelial cell antibody was 1:10. Providing the clinical symptoms and laboratory results, she was diagnosed with Behçet’s disease. She was prescribed Methylprednisolone 8 mg qd and traditional Chinese medicine. While the old nodosum could still be seen vaguely, only a single new oral ulcer and no new erythema nodosum appears 6 months later, and the physician told her to stop steroid therapy and add Thalidomide 50 mg qn. However, she developed a morning stiffness which she could recover by herself a few minutes later. Ultrasound showed no malformation or swelling of the involved joints (wrists and elbows), and rheumatoid factor was negative. Besides, she developed lethargy which she thought was the side effect of Thalidomide. Thus, Thalidomide was stopped and Hydroxychloroquine 200 mg bid was added.

**Figure 1.**
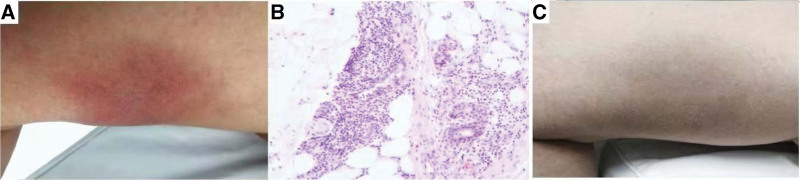
(a) EN lesions prior low-dose IL-2. (b) Neutrophil and lymphocyte infiltrate in the dermis (hematoxylin-eosin, original magnification × 400). (c) EN lesions after treatment with low-dose IL-2. IL-2 = interleukin-2.

However, 1 month later, several ulcers reappeared in the buccal and labial mucosal membranes, with a 9/10 visual analogue scale evaluation. In addition, there is an erythema nodosum above the front of the right leg after trauma. By inspection, a dark red plague emerged on the lower part of the right leg. The pathology shows panniculitis which could arise from erythema nodosum, and immune pathology and tuberculosis were negative. Also, the pathergy test is positive for her. A diagnosis of Behçet’s disease was further confirmed with the exclusion of other complications. Given the situation of this patient, we decide to add low-dose IL-2 qod for 3 months. After the first month of injection of IL-2, the number of her oral ulcer decreased from 3 to 0, though the erythema nodosum still exists. Surprisingly, it completely disappears right before she stops IL-2 after 3 months, and she thinks visual analogue scale evaluation keeps reducing to 0 (Fig. 1a–c). At the time of writing of this case report, the patient had been symptom-free for 6 months. No adverse side effects were observed during IL-2 administration.

**Figure 2.**
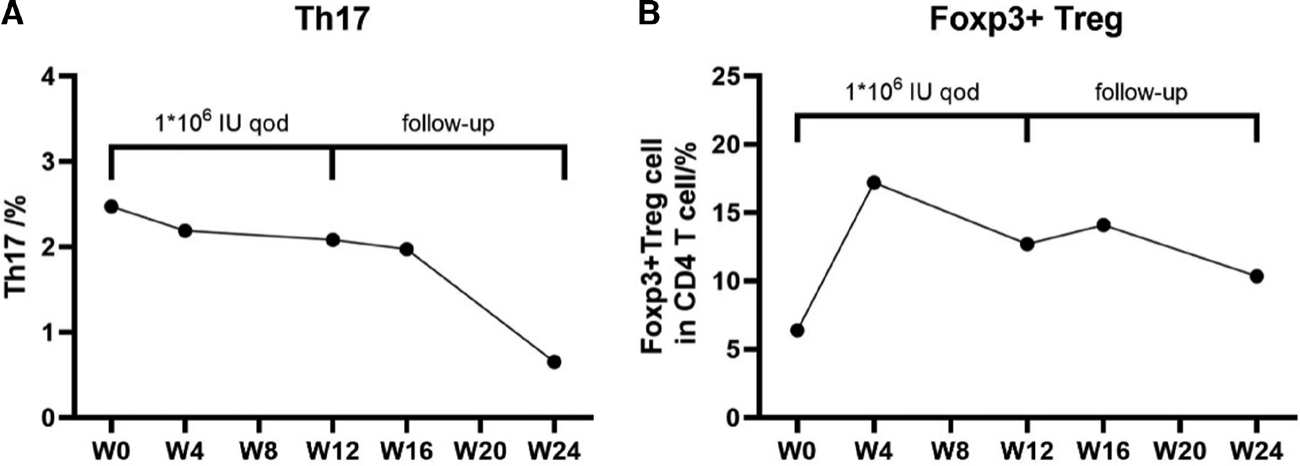
Treg (a) and Th17 (b) cell levels prior to initiation of treatment with interleukin-2 (IL-2) (2021.6.23), with treatment with IL-2 106 IU administered once every other day for 3 months (2021.6.24–2021.9.28) and cease all medicine for 3 months (2021.9.29–2021.12.7). IL-2 = interleukin-2.

**Figure 3.**
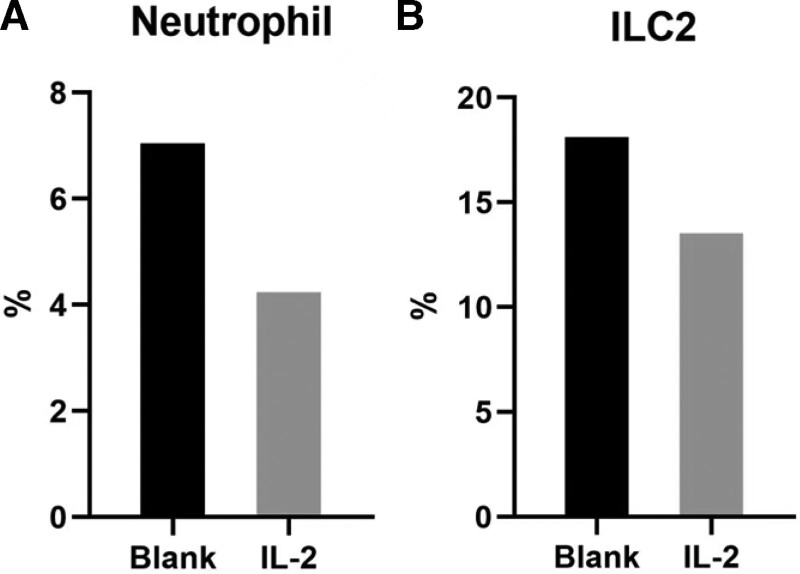
Neutrophil (a) and IL-2 (b) cell levels of peripheral blood in vitro. We extracted lymphocytes, applied NS 10 μL to the blank and IL-2 10 μL to the other, and incubated in a 36°C incubator for 3 days. IL-2 = interleukin-2.

Treatment with low-dose IL-2 resulted in an increase in Foxp3 + Treg cells and a decrease in Th17 (Fig. 2a and b). Before she was treated with IL-2, we acquired her blood for test in vitro by her permission. We treated the peripheral blood mononuclear cell with IL-2 (10 μL), incubated in a 36℃ incubator for 3 days and tested neutrophil and ILC2 subtype cells. Compared to the blank group (NS 10 μL), both of them decrease after the application of IL-2 (Fig. 3a and b).

## 3. Discussion

We report a patient diagnosed with Bechet’s disease based on the international criteria of Bechet’s disease.^[9]^ The patient was successfully treated with low-dose-IL-2, which improved the clinical manifestation and relieved the ache.

Treg cells are essential for suppressing the activation and infiltration of an excessive immune and inflammatory response, while neutrophil acts in the opposite direction.^[10]^ Neutrophil hyperfunction plays a significant role in the mechanisms of Behcet syndrome. The immune analysis patient of the patient shows a two-fold increase in Foxp3 + Treg and a slight steady decrease in Th17 after application of the medicine. The in vitro experiment also illustrates the decline in neutrophil and ILC2. Thus, after the treatment, the patient obtains remission in both aspects of clinical symptoms and immunological cell levels.

The patient had a poor response to Thalidomide and topical corticosteroids. Providing cytotoxicity which immunosuppressants can bring, the low-dose IL-2 can be considered a new-type and effective treatment for Behçet’s disease and is relatively safer. The safety of low-dose IL-2 has been shown in several previous studies.^[8]^ It is interesting to note that no serious side effect of low-dose of IL-2 has been reported.

## Acknowledgements

We acknowledge the patients, research nurses and clinicians who have helped support this study.

## Author contributions

**Conceptualization:** Jing He.

**Data curation:** Wenyan Zhou, Tian Liu.

**Formal analysis:** Wenyan Zhou, Xian Xiao.

**Investigation:** Tian Liu, Xian Xiao.

**Supervision:** Jing He.

**Writing – original draft:** Wenyan Zhou.

**Writing – review & editing:** Wenyan Zhou, Tian Liu, Jing He.
